# Intelligibility in Context Scale: validity of the Brazilian-Portuguese version

**DOI:** 10.1590/2317-1782/e20240138en

**Published:** 2025-02-24

**Authors:** Matheus Francoy Alpes, Julia Cipolato, Ana Rita Valente, Jacqueline Aquino do Nascimento, Patrícia Pupin Mandrá, Marisa Lousada

**Affiliations:** 1 Instituto de Saúde de Nova Friburgo, Universidade Federal Fluminense – UFF - Nova Friburgo (RJ), Brasil.; 2 Faculdade de Medicina, Universidade de São Paulo – USP - Ribeirão Preto (SP), Brasil.; 3 Escola Superior de Saúde, Universidade de Aveiro - Aveiro, Portugal.; 4 Faculdade de Odontologia, Universidade de São Paulo – USP - Bauru (SP), Brasil.

**Keywords:** Speech Intelligibility, Validation Study, Child Language, Speech, Language and Hearing Sciences

## Abstract

**Purpose:**

The aim of the study was to validate the Brazilian-Portuguese version of the Intelligibility in Context Scale (ICS-BP).

**Methods:**

Sixty children (mean age in months = 55.05, SD = 4.2), 15 with parental or teacher concerns regarding their speech and 45 without concerns, underwent assessment using the phonology subtest of ABFW – Child Language Test. The percentage of consonants correct (PCC) was calculated and parents completed the ICS to evaluate their children's intelligibility with various communication partners. Descriptive statistics were obtained. Kruskal-Wallis and Spearman’ tests were used for independent group comparisons and correlation calculations, respectively. Linear regression models were established to predict PCC. Internal consistency was calculated using Cronbach's alpha. A Receiver Operating Characteristic (ROC) was used to analyse sensitivity and specificity. Significance was considered for p-values under 0.05.

**Results:**

The majority of parents reported a mean score of 4.6 in a total of 5 (SD .10) at the ICS responses with different listeners, with better performance with other acquaintances and members of the family. The ICS demonstrated excellent internal consistency (α = 0.95). Positive correlations were found between ICS scores and PCC (r = .790) and a simple linear model was established between the ICS mean score and PCC. Sensitivity (0.98) and specificity (0.87) were considered high.

**Conclusion:**

The ICS-BP indicated high psychometric values, suggesting that this instrument can be used to measure the intelligibility of Brazilian children.

## INTRODUCTION

Language plays a fundamental role in human interaction with the environment, allowing the individual to structure their thoughts, translate what they feel, express what they already know, and communicate with others^(1,2)^.To achieve this, the message needs to be clear, cohesive, objective, and intelligible to the listener^(3)^.

The concept of speech intelligibility can be understood as how the interlocutor's speech is understood by the listeners^(3)^. Phonological skills of Brazilian portuguese speaking children tend to increase with age, observing an accelerated process in the acquisition of language phonemes between two and four years of age, when their speech becomes intelligible^(4,5)^.

Intelligibility is usually impaired in children with speech sound disorder^(6)^. The speech of children with speech sound disorders is characterized by a restricted phonetic inventory and several phonological processes^(7)^ that can lead to unintelligible speech, causing problems in the act of communicating^(8)^. These demands can be evidenced by instruments validated for this purpose (e.g., Intelligibility in Context Scale - ICS).

This severity can be analyzed quantitatively, based on the calculation of the Percentage of Consonants Correct (PCC) generating a scale of different degrees of speech impairment^(9)^. A lower PCC score is correlated with a greater impact on speech intelligibility^(10)^.

Furthermore, a qualitative assessment of the communicative pattern carried out based on the perception of the child's most common communicative partners, such as family members, school staff or pediatricians, becomes essential for a better understanding of the child's communicative profile^(11,12)^. Although different methods are available, few scales have been studied for their application with this population.

The ICS is a scale that has been validated with the Australian children population^(13)^. The ICS requires parents to estimate a child’s speech understandability in a range of environmental contexts and by different listeners (immediate family, extended family, friends, acquaintances, teachers, and strangers/unfamiliar people) on a five-point scale (1 = never, 2 = rarely, 3 = sometimes, 4 = usually, 5 = always).

The ICS has been translated into more than 60 languages such as the European-Portuguese - Escala de Inteligibilidade em Contexto (ICS-EP)^(14)^ and Brazilian-Portuguese - Escala de Inteligibilidade em Contexto (ICS-BP)^(15)^. Furthermore, several psychometric validation studies have already been carried out in different contexts as Vietnamese^(16)^ and Chinese^(17)^ with high-sensitivity and specificity values indicating that ICS could be used as a screening tool to identify children who require additional evaluation for speech sound disorders.

In the European Portuguese study, seventy-six children, 25 with parental or teacher concerns about the way they spoke and 51 without concerns, were assessed through the use of percentage of correct phonemes (PPC), percentage of correct consonants (PCC) and percentage of correct vowels (PVC). The ICS was then completed by parents to estimate their children's intelligibility with different communicative partners. The results showed that item-level scores were different according to the communicative partners. High values ​​were obtained for sensitivity (0.80) and specificity (0.84), using a cutoff point of 4.36. ICS-EP presents good psychometric properties, suggesting that it is a valid tool for estimating the intelligibility of children when they talk to different communicative partners, and this version of the ICS can be used as a screening measure of the speech intelligibility of Portuguese children^(14)^.

In Brazil, research on this topic is scarce. Studies carried out with children in the process of language acquisition and development showed that in narratives and naming, all correlations between intelligibility and severity of speech sound disorders were strong and directly proportional. It was found that the more intelligible the speech of the child, the milder the severity of the disorder was classified^(18,19)^.

However, no studies were found on the psychometric validity of the Brazilian-Portuguese version. Thus, this study aims to analyze the psychometric properties of the ICS-BP, specifically internal consistency, criterion validity, sensitivity, and specificity.

## METHODS

### Participants

All individuals involved (or their guardians) signed the Informed Consent Form (ICF). All children were recruited and assessed by Speech and Language Pathologists (SLPs) at a single school (following the suggestion of the Ethics Committee). This study comprised sixty children, with 15 exhibiting parental/teacher concerns about their speech and 45 without identified concerns. None of the children had any biomedical conditions or persistent hearing impairments, although 4 caregivers (6.6%) mentioned a history of ear infections (information obtained through the parental questionnaire). All participants demonstrated normal-range nonverbal intelligence (>25th percentile) on the Brazilian version of Raven's Coloured Progressive Matrices^(20)^. Brazilian Portuguese served as the native language for all participants. The socioeconomic level (refer to Table 1) was determined by cross-referencing the ABEP indicator, following the Brazil Standard Economic Classification Criteria^(21)^.

**Table 1:** Sociodemographic and sample characterization

**Variables**	N (%)		N (%)
**Gender**	46 (76.7)	**Socio-economic status**	10 (16.7)
Male	14 (23.3)	High	48 (80)
Female		Medium	2 (3.3)
		Low	
**Age (months)**	20 (33.3)	**Ear infection history**	4 (6.7)
**49-53**	25 (41.7)	Yes	56 (93.3)
**54-59**	15 (25)	No	
**>60**			

Descriptive statistics were presented in terms of counts and percentages

The Ethics Committee ensured all ethical procedures (reference number 1.449.191).

### Sociodemographic and sample characterization

All children in the study age group enrolled at the school were invited to participate. Among the 60 children who participated in the study, more of them were male (n = 46, 76.7%). The children’s ages ranged from 49 to 60 months (M = 55.05, SD = 4.2). The majority of the sample (n = 48, 80%) presented a medium socioeconomic status (see Table 1).

### Tools

#### Intelligibility in Context Scale (ICS)

The ICS is a seven-item parent-rated measure of children’s intelligibility when communicating with people with different levels of familiarity and authority, using a five-point Likert scale. For this study, the ICS-BP was used^(15)^. The translation has been undertaken before by one SLP and researcher who works with children with speech sound disorders and is a native speaker of Brazilian Portuguese.

#### Child Language Test (ABFW)

Phonological system assessment validated with the Brazilian children population, using part A (naming of 34 figures with different phonemes from Brazilian Portuguese)^(22)^. The test is indicated for children aged between 2 and 6 years, with application time varying depending on the age and specific characteristics of each child. In the naming test, the SLP asked each individual to say the name of the figure shown. The figures were presented in front of the individuals enabling a clear vision. After the application, the Percentage of Consonants Correct – PCC^(9)^ was calculated.

#### Questionnaire for parents

Caregivers filled out a questionnaire designed to provide information about the children, including the absence of any biomedical conditions, native language, and a history of ear infections. Additionally, the questionnaire aimed to gather details about the family background and stated that their children had, or did not, have difficulties in being understood when expressing themselves specifically regarding socioeconomic status. A specific query in the questionnaire asked caregivers, “Do you have any concerns about how your child talks and makes speech sounds?” with three response options: yes, a little, or no, as in previous ICS studies^(14)^.

### Procedure

#### Recruitment

Children from a particular kindergarten underwent screening through assessments provided by parents and teachers to identify those facing challenges in verbal communication and speech sound production. Subsequently, 60 children, comprising 15 identified by their parents and teachers as experiencing speech difficulties and another 45 without such identification, underwent a comprehensive evaluation conducted by both a speech-language therapist and a psychologist. The ICS can be accessed at http://www.csu.edu.au/research/multilingual-speech/ics.

#### Assessment

All children underwent evaluations conducted by two pediatric speech-language therapists. These one-hour sessions occurred in a tranquil space within their respective kindergarten or childcare centers. No hearing assessment was carried out on the children considering that the authors used the same procedures reported in the ICS validity study for European portuguese^(14)^. Following approval from the children and their adult guardians, the assessments were audio-recorded using Audacity software on a laptop so that they could be analyzed later. ABFW - phonology subtest was employed to evaluate the phonology skills of all children. Examiners recorded phonetic transcriptions online, and the audio files were reviewed two days post-assessment to verify the accuracy of the transcriptions. Two trained transcribers did independent phonetic transcriptions of the sample; consensus was reached through discussion, with any discrepancies resolved through discussion. Parents completed a questionnaire and the ICS during their child's assessment.

#### Data analysis

Descriptive statistics were presented in terms of mean (M), standard deviations (SD), median (Med), and percentiles (P25% and P75%) for continuous variables, while counts and percentages were used for categorical variables. The comparison of two or more independent groups was conducted using the Kruskal-Wallis test. Spearmans’ test was utilized for correlation calculations. Linear regression models were established to predict severity measure (PCC), with regression ANOVA testing slope significance and residual normality confirmed via visual inspection of the PP plot. The internal consistency of the ICS was evaluated using Cronbach's alpha. Sensitivity and specificity were assessed through a Receiver Operating Characteristic (ROC) analysis based on ICS and parental opinion, calculating the area under the curve (AUC) and corresponding 95% confidence interval (CI). Statistical analyses were performed using SPSS® Software, version 29.0 (SPSS Inc., Chicago, IL), and significance was considered for p-values under 0.05.

## RESULTS

The influence of demographic variables (gender, age, and socioeconomic status) on ICS scores was examined (Table 2). No significant differences were observed in ICS scores based on gender (p = 0.456), age (p = 0.142) or socioeconomic status (0=0.743).

**Table 2:** ICS and PCC

	**ICS**	**PCC**
	M±SD P25;Med;P75	M±SD P25;Med;P75
**Gender**		
Male (N = 46)	4.58±0.59 4.39;4.86;5.00	92.93±7.75 89.75;92.00;100.0
Female (N = 14)	4.71±0.63 4.71;4.93;5.00	95.79±8.62 93.50;100.0;100.0
Statistical result	X^2^(1)=0.555; p=0.456	X^2^(1)=2.238; p=0.135
Effect size	0.01	0.04
**Age**		
<53 (N = 20)	4.67±0.66 4.71;4.93;5.00	94.25±9.22 89.25;100.0;100.0
54-59 (N = 25)	5.00±0.46 4.57;5.00;5.00	93.80±6.72 90.0;92.00;100.0
>60 (N = 15)	4.39±0.68 4.00;4.71;5.00	95.00±8.57 89.0;95.0;100.0
Statistical result	X^2^(1)=3.908; p=0.142	X^2^(1)=1.087; p=0.581
Effect size	0.07	0.02
**Socioeconomic status**		
High (N = 10)	4.48±0.77 4.00;4.78;5.00	93.00±9.56 89.25;96,00;100.0
Medium (N = 48)	4.67±0.52 4.60;4.86;5.00	93.92±7.50 90.0;98.0;100.0
Low (N = 2)	4.00±1.41 3.00;4.00	89.00±15.55 78.00;89.00
Statistical result	X^2^(1)=0.594; p=0.743	X^2^(1)=0.594; p=0.743
Effect size	0.01	0
**Parents evaluation**		
With identified concern (N = 15)	3.80±0.71 3.14;4.00;4.29	85.73±9.30 78.0;90,0;92.0
No identified concern (N = 45)	4.88±0.15 4.71;5.00;5.00	96.22±5.46 91.00;100.0;100.0
Statistical result	X^2^(1)=28.577; p<.001	X^2^(1)=14.224; p<.001
Effect size	0.48	0.24

The comparison of two or more independent groups was conducted using the Kruskal-Wallis test

The ICS results using a five-point Likert scale (1 = never, 2 = rarely, 3 = sometimes, 4 = usually, 5 = always) are outlined in Table 3. The overall mean total score for the entire sample was 4.61 (SD = .10). Analysis of mean scores for the seven ICS items revealed variations in parental ratings based on the communication partner: acquaintances (M = 4.70), family members (M = 4.68), extended family members and teachers (M = 4.67), friends (M = 4.65), parents (M = 4.55), and strangers (M = 4.42). Parental responses ranged from *rarely* (2) to *always (5)*, with no response to *never* (1) in all items.

**Table 3:** Ratings for the ICS items (N = 60)

Item	Mean ± SD		Always (5)		Usually (4)		Sometimes (3)		Rarely (2)		Never (1)	
With identified concern	No concern	With identified concern	No concern	With identified concern	No concern	With identified concern	No concern	With identified concern	No concern	With identified concern	No concern
Do you understand your child?	4.07 ± (0.70)	4.71 ± (0.46)	4 (26.7%)	32 (71.1%)	8 (53.3%)	13 (28.9%)	3 (20%)	0	0	0	0	0
Do immediate members of your family understand your child?	4.07 ± (1.03)	4.89 ± (0.32)	7 (46.7%)	40 (88.9)	3 (20%)	5 (11.1%)	4 (26.7%)	0	1 (6.7%)	0	0	0
Do extended members of your family understand your child?	3.67 ± (0.90)	5 ± (0)	2 (13.3%)	45 (100%)	8 (53.3%)	0	3 (20%)	0	2 (13.3%)	0	0	0
Do your child’s friends understand your child?	3.73 ± (0.70)	4.96 ± (0.21)	2 (13.3%)	43 (95.6%)	7 (46.7%)	2 (4.4%)	6 (40.0%)	0	0	0	0	0
Do other acquaintances understand your child?	3.93 ± (0.80)	4.96 ± (0.21)	4 (26.7%)	43 (95.6%)	6 (40.0%)	2 (4.4%)	5 (33.3%)	0	0	0	0	0
Do your child’s teachers understand your child?	3.73 ± (0.70)	4.98 ± (0.15)	2 (13.3%)	44 (97.8%)	7 (46.7%)	1 (2.2%)	6 (40.0%)	0	0	0	0	0
Do strangers understand your child?	3.47 ± (0.74)	4.73 ± (0.45)	1 (6.7%)	33 (73.3%)	6 (40.0%)	12 (26.7%)	7 (46.7%)	0	1 (6.7%)	0	0	0

Significant correlations were identified among seven items on the ICS through bivariate nonparametric correlation analysis (Spearman’s rho), with correlation coefficients ranging from rho = .41 to rho = .100, p = .001. The weakest correlation emerged between parents and acquaintances (rho = 0.48). The internal reliability of the ICS was assessed using Cronbach’s alpha (α = 0.95), indicating a robust internal consistency - Table 4.

**Table 4:** Inter-item correlations for the ICS (N = 60)

Item	1. Parent	2. Immediate family	3. Extended family	4. Child’s friends	5. Acquaintances	6. Teachers	7. Strangers
1. Parent	1	0.65	0.62	0.62	0.54	0.60	0.68
2. Immediate family		1	0.81	0.73	0.78	0.75	0.58
3. Extended family			1	0.91	0.90	0.93	0.80
4. Child’s friends				1	0.86	0.98	0.79
5. Acquaintances					1	0.87	0.76
6. Teachers						1	0.78
7. Strangers							1

Spearmans’ test was utilized for correlation calculations

The criterion validity of the ICS was examined in a sample of 60 children, with 15 whose caregivers expressed concerns about their speech and 45 without any reported concerns. In this study, the ICS was juxtaposed with the participant's PPC derived from data obtained from the ABFW. Bivariate correlation analysis using Spearmans’ rho revealed a positive correlation between the mean score of the ICS and PCC (rho = .793, p <001) - Table 5.

**Table 5:** Correlation Analyses ICS and PCC

		*PCC*	*ICS*
*Spearman*	PCC	1.000	.793
*Correlation*	ICS	.793	1.000
*Sig.*			<001

Spearmans’ test was utilized for correlation calculations

**Figure 1:**
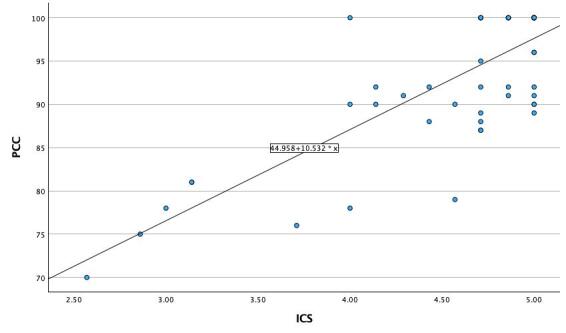
Simple Linear Regression

Linear regression model is presented in Figure 1. The proportion of variability explained was 62,8%.

**Figure 2:**
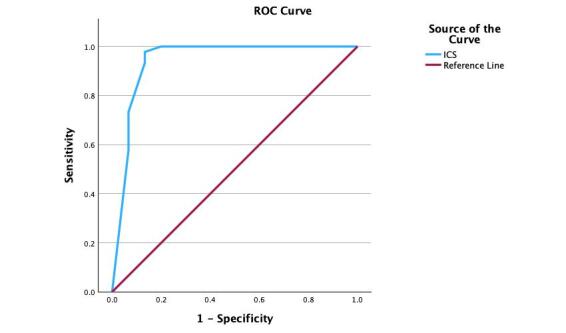
The Receiver Operating Characteristic (ROC)

Sensitivity and specificity were evaluated using a Receiver Operative Characteristic (ROC) based on ICS, as presented in Figure 2. The values of sensitivity (0.98), specificity (.0.87), and AUC (0.941) were high and considered good. The cut-point score for the sensitivity and specificity levels was 4.5.

## DISCUSSION

The objective of this study was to validate the Brazilian version of the ICS using a sample of 60 parents of Brazilian children aged between 49 to 60 months. The potential impact of sociodemographic factors such as gender, age, and socioeconomic status on the mean scores of the ICS was examined, revealing no significant differences.

The investigation involving English-speaking children revealed substantial gender differences^(23)^. In contrast, other studies^(14,16)^ did not find significant results. This disparity in findings may be attributed to variations in sample sizes employed across the studies. Regarding the age variable, the study of European-Portuguese^(14,)^ and German^(22)^ found significant differences between the groups of younger and older children, not corroborating the data obtained in this study. Regarding socioeconomic status, there was a predominance of children with medium status (80%), a fact that can be explained by their being from a private kindergarten, concerning results by other studies^(14,24)^.

Studies that validated the ICS around the world found a mean score of 4.4 (European-Portuguese children: 4.6; German children: 4.4). This data shows that the majority of children in this study presented a mean score of 4.6, represented by “usually” to “always” (4 or 5) at the ICS responses with different listeners, with better performance with other acquaintances, members of family, extender members of family, teachers, friends, parents and lastly strangers. This fact reaffirms the importance of the environment for the language acquisition and development process^(25)^ and the fact that most Brazilian children spend most of the day at school or in the care of other caregivers, their regular listeners^(26)^. Furthermore, it is already known that the family plays a very important role in the constant development of children's general skills including language, and recognizing possible signs of delay or difficulties is of paramount importance for an earlier assessment and intervention process^(27)^.

To check internal consistency, Cronbach's alpha was calculated, which normally scores between 0 and 1 with a minimum acceptable value of 0.70^(28)^. The value obtained in this study was α = 0.95, demonstrating a value classified as very high. This data is very similar to that obtained in the original study (α = 0.93)^(23)^ and the Portuguese study (α = 0.96)^(14)^.

Criterion validity was established through significant correlations between the ICS and PCC (rho = .79). This result is higher than the Portuguese study (r = .65) and the Vietnamese study (r = .42). The proportion of variability explained in the linear regression model was 62,8%, showing fit to the studied sample.

The values obtained for sensitivity were above 0.98, similar to other studies (European Portuguese: 0.80; Jamaican: 0.84; German: 0.90). This data suggests that the ICS can be used as a screening scale to identify language demands.

The Portuguese language (spoken in Brazil and Portugal) has some differences at various levels as semantic, morphosyntactic, phonetic/phonological, and also prosodic, including the intelligibility of speech^(29)^. Despite this, the ICS validation study with Portuguese children showed very similar results to the study carried out with Brazilian children, as described in this excerpt.

Brazilian studies about speech intelligibility also showed positive correlations between the severity of speech sound disorders and presented SLPs with greater effectiveness in judging the speech intelligibility of children that have speech sound disorders compared to the nonprofessional group^(18,19)^.

The study presents an important contribution to the area of ​​child language and speech pathology in Brazil, with the validation of a screening scale that can be used with family members by health or education professionals to identify possible language demands in childhood. However, some limitations need to be highlighted, such as the number and characterization of the sample and a single school for data collection. Therefore, the importance of continuing research that checks other variables and characterizes a sample completely is indicated, thus enabling the determination of other associated psychometric properties.

## CONCLUSIONS

The ICS-BP indicated high psychometric values, suggesting that this instrument can be used to measure the intelligibility of Brazilian children.

However, the fact that the sample comes from just one preschool may be a limitation of the study, which should be extended to other realities.
